# Prevalence of Dental Caries in Children in Mymensingh and Its Associated Risk Factors: A Cross-Sectional Study

**DOI:** 10.3390/dj10070138

**Published:** 2022-07-20

**Authors:** Sharmin Sultana, Mst. Sonia Parvin, Md. Taohidul Islam, Emdadul Haque Chowdhury, A. S. Mahfuzul Bari

**Affiliations:** 1 Department of Pathology, Faculty of Veterinary Science, Bangladesh Agricultural University, Mymensingh 2202, Bangladesh; sharminbds@gmail.com (S.S.); bari.bau.bd@gmail.com (A.S.M.B.); 2 Population Medicine and AMR Laboratory, Department of Medicine, Faculty of Veterinary Science, Bangladesh Agricultural University, Mymensingh 2202, Bangladesh; soniaparvin@bau.edu.bd (M.S.P.); taohid@bau.edu.bd (M.T.I.)

**Keywords:** dental caries, Mymensingh, prevalence, risk factors

## Abstract

Background: Children suffer from various oral and periodontal diseases. Dental caries is one of the most prevalent oral diseases among children in the world. This study was conducted to identify the prevalence and risk factors of dental caries in children in Mymensingh, Bangladesh. Methods: A cross-sectional study was conducted on 362 pediatric patients who attended the Dental Unit of Mymensingh Medical College from March to September 2019. The sample size was calculated using a statistical formula and the children were selected using a systematic random sampling technique. Children and their guardians were interviewed and data were recorded using a structured questionnaire. Risk factors were analyzed using multivariate logistic regression. Results: The overall prevalence of dental caries was 82.7%. The prevalence of caries was significantly higher in aged children (8–10 years) and also in rural, low-income, and illiterate families. Seven significant risk factors were identified that included residence in the rural area (OR: 7.31 [1.73–30.83]), a parental income of BDT ≤ 20,000 per month (OR: 4.75 [1.49–15.05]), reduced duration (≤1 min) of teeth cleaning (OR: 18.54 [2.05–168.17]), teeth cleaning before breakfast (OR: 93.30 [10.95–795.32]), the spoon-feeding method (OR: 12.57 [2.09–75.61]), long-term (37–48 months) breastfeeding (OR: 212.53 [8.69–5195.25]), and family oral problem (OR: 8.20 [2.57–26.16]). Conclusions: The prevalence of dental caries among the children in Mymensingh is very high and was associated with residence in rural areas, parental income, reduced duration of teeth cleaning, teeth cleaning before breakfast, the spoon-feeding method, long-term breastfeeding, and family oral problems.

## 1. Introduction

Oral health is a part of a person’s health and general wellbeing and is considered very important for a good and consistent quality of life. The teeth are one of the most important parts of the oral cavity. They not only add to the beauty of the face but also help digest food. Moreover, they enable words to be articulated and pronounced correctly [1].

Poor oral health influences oral diseases. The experience of pain, and problems with eating, chewing, smiling, and communication due to missing, discolored, and damaged teeth impacts people’s daily lives and wellbeing. Oral diseases also restrict activities at school, office, and at home. The most common oral diseases are dental caries, loss of teeth, oral or oropharyngeal cancer, mouth sores, congenital anomalies, and other disorders, such as human immune-deficiency syndrome (HIV/AIDS)-related oral disease (WHO, 2007). Among these, dental caries is the most common oral disease, particularly in pediatric patients [2,3,4,5,6]. It not only negatively affects the masticatory function but also blocks the intake of nutrients and harms the growth of permanent teeth, which can damage the oral mucosa and impact general health and quality of life [7]. The *Global Burden of Disease Study 2017* estimated that oral diseases affect close to 3.5 billion people worldwide, with caries of permanent teeth being the most common condition [8]. Globally, it is estimated that 2.3 billion people suffer from caries of permanent teeth and more than 530 million children suffer from caries of primary teeth [9,10,11].

Dental caries is increasing in both developed and developing countries [12,13,14,15,16,17]. It has been reported that the prevalence of caries ranges up to 12% in developed countries [18], whereas in less developed countries, particularly among disadvantaged groups, the prevalence has been reported to be as high as 70% [19,20]. A high prevalence of caries in children has also been reported in some developed countries such as the United Arab Emirates (83.0%), Greece (64.0%), Brazil (45.8%), Israel (64.7%), China (85.0%), South Africa (49%), and Great Britain (39.4%) [21,22,23,24,25,26,27,28].

In Bangladesh, dental caries is a common oro-dental problem [29,30,31,32,33]. Above 40% of children under 5 years old suffer from dental caries [34,35,36]. It has been reported that approximately 88% of people between 13–22 years old are missing one or more permanent teeth due to dental caries or related complications [37,38].

Dental caries develops in the presence of fermentable carbohydrates, a bacterial substrate, and a susceptible tooth surface [39]. Various contributory risk factors responsible for the development of dental caries are ignored due to illiteracy, a low family income, the inadequate practice of oral hygiene, dietary factors, such as the consumption of sweeteners, fluoride in the water, and socioeconomic factors [12,40,41,42,43,44,45].

Regular teeth cleaning is an important factor to prevent oral diseases. Limited awareness about oral health and poor knowledge of oral hygiene habits prevails among children from 5 to 12 years of age [46,47,48]. Several studies report that the relationship between age and practicing oral hygiene is statistically significant [49,50]. Oral hygiene education programs significantly reduce the prevalence of dental caries in a population [33].

The prevalence and risk factors associated with dental caries are of great interest, but data related to these in pediatric patients are limited in Bangladesh. A limited survey on the prevalence of oral diseases was conducted, but the risk factors associated with dental caries in children remain unknown. Therefore, the objectives of the study were to estimate the prevalence and identify the associated risk factors of dental caries in children in Mymensingh in Bangladesh.

## 2. Materials and Methods

### 2.1. Sample Size and Selection of Children

A cross-sectional study was conducted on 362 children from 4 to 10 years old, who were registered at the Outdoor Dental Unit of Mymensingh Medical College Hospital (MMCH) from March to September 2019. A total of 1850 children were registered during the study period. The sample size was calculated using the formula *n* = Z^2^ × *p* (1 − *p*)/d^2^, where *n* = number of children, Z = 1.96 (standard normal variant at 95% CI), d = 5% (absolute error or precision), and *p* = 62%, the expected prevalence [51]. Thus, the estimated sample size was 362 children. A systematic random sampling technique was used to select the children [52]. A number between one and ten was randomly selected, which was seven, and then every *7th* patient from 10 children aged 4–10 years old with dental problems that registered at Outdoor Dental Unit of MMCH, was included in the study. Informed written consent was obtained from the parents or legal guardians. Before obtaining consent, the objectives of the study along with the procedure, risks, and benefits were explained to the parents or legal guardians using easily understandable local language. However, children with any systemic diseases, such as kidney, heart, or liver diseases, were excluded. Furthermore, children with any acute infections, fever, or diarrhea during the clinical examination or difficulty in opening the mouth were also excluded. Seven children were excluded because one had diabetes, one had a fever, and five had patent foramen ovale. Until the estimated sample size was attained, the selection continued.

### 2.2. Ethical Consideration

The study was approved by the Ethical Standard of Research Committee of the Bangladesh Agricultural University Research System (BAURES/ESRC/VET/15-1, dated 28 February 2019). In addition, written permission was obtained from the Head of the Dental unit and the Director of Mymensingh Medical College Hospital, Mymensingh. Informed written consent for data collection was obtained from the parents or legal guardians. Before obtaining consent, the objectives of the study along with the procedure, risks, and benefits were explained to the parents or legal guardians using easily understandable local language. All methods were performed in accordance with the relevant guidelines and regulations.

### 2.3. Clinical Examination and Data Collection

The clinical examination of the children was performed under head lamp illumination using a disposable dental mirror and ball-ended community periodontal index (CPI) probe by the researcher herself, who is a registered experienced dental surgeon. Food debris was gently removed to avoid under-recording of dental caries. After clinical examination, data on sociodemographic characteristics, oral hygiene practices, eating habits, and dietary habits were collected from the parents or legal guardians and the children through face-to-face interviews.

### 2.4. Diagnosis of Dental Caries

The criteria recommended by the WHO were followed for the diagnosis of dental caries [53]. Caries is a localized dissolution and destruction of calcified tissues of the teeth and appears black in color. Briefly, caries experience was measured with the *dmft* index. A tooth was recorded as decayed (dt) when a lesion had an unmistakable cavity, undermined enamel, or detectably softened floor or wall. If a child had at least one decayed tooth, it was considered a case of caries.

### 2.5. Statistical Analysis

Descriptive statistics was performed to calculate the prevalence of dental caries or tooth decay. The Chi-square test (Z-test for proportion) was performed to find out the significant deference in the prevalence between different exposure variables. Bivariable followed by multiple logistic regression (forward stepwise selection) analysis was performed to identify the associated risk factors of dental caries related to sociodemographic characteristics, and dietary and oral hygiene practices. The variables with a *p*-value of ≤0.3 in bivariable logistic regression were entered into the multiple regression analysis. The correlation (multicolinearity) among independent variables was checked during model building. The level of significance was set at ≤0.05. All analyses were performed using the statistical package for social sciences (SPSS) version 22.0 (IBM Bangladesh Pvt. Ltd., Dhaka 1000, Bangladesh).

## 3. Results

### 3.1. Prevalence of Dental Caries

The overall prevalence of dental caries was 82.7%, of which 83.8% were in male and 81.8% in female children (Table 1). The caries prevalence was comparatively higher in 8–10-year-old children (85.4%) than 4–7-year-olds (82%). The prevalence was significantly higher in children from rural areas (96.3%) than those from suburban (83.9%) and urban (68.9%) areas. Around 95% of children from low-income families had dental caries, which was significantly higher than children from high-income families (65.5%). All children who had illiterate mothers were affected with dental caries.

Dental caries prevalence in children categorized by oral hygiene practices is shown in Table 2. The children who cleaned their teeth twice or more a day had a significantly lower prevalence (67.4%) of dental caries than those who cleaned their teeth once a day (87.8%). Furthermore, the lowest occurrence (11.1%) was observed in children who cleaned their teeth after breakfast and before they slept at night. The highest occurrence of dental caries (96%) was observed in children who spent less than one minute to clean their teeth. A very high prevalence (90.2%) was recorded in children whose parents did not have any knowledge about oral hygiene. A very high prevalence (93.2%) was also observed in children whose families had a history of oral problems compared to those whose families did not have a history of oral problems.

The occurrence of dental caries related to dietary and eating habits is summarized in Table 3. The prevalence of dental caries was 82.1% and 87.5% in children who had a habit of bottle and spoon feeding, respectively, during their early life. The children who had a habit of drinking milk with sugar experienced a significantly higher occurrence (90.8%) of dental caries than those who drank milk without sugar (63.0%). The prevalence of dental caries was significantly higher in children (92.2–98.9%) who breastfed for a long time (≥25 months) than those who breastfed for a short time (0–24 months). There was a significantly higher prevalence of caries in children who had the habit of chewing gum (87.6%) compared to those who did not (69.8%). However, a lower occurrence of dental caries was observed in children who had the habit of eating chocolate and chips and drinking soft drinks as compared with those who did not have such habits, though the differences were not statistically significant.

### 3.2. Risk Factors of Dental Caries

In the bivariable analysis, among the 39 variables (seven sociodemographic factors and thirty-two variables related to dietary and oral hygiene practices), 26 were significantly associated with the occurrence of dental caries. Of them, five were sociodemographic factors and twenty-one were related to dietary and oral hygiene practices. Sociodemographic factors such as residence in rural areas, religion, parental occupation, parental income of ≤20,000.00 BDT per month, and maternal education (secondary school certificate) had the strongest point estimate and statistical significance (*p* < 0.001) (Table 4). Factors related to dietary and oral hygiene practices such as teeth cleaning material (tooth powder), frequency of teeth cleaning (once a day), teeth cleaning before breakfast, parents without oral hygiene knowledge, frequency of drinking soft drinks (everyday), habit of chewing gum, habit of drinking milk with sugar, spoon feeding in early life, habit of breastfeeding, long-term (≥25 months) breastfeeding, and parental caries had the strongest point estimate and highest statistical significance (*p* < 0.001) (Table 5).

In multiple logistic regression, among the 29 variables (with *p* ≤ 0.3 in the bivariable analysis), 24 were included. Of these, seven variables were identified as the potential significant risk factors (Table 6). The risk factors were residence in rural areas, parental income of ≤20,000.00 BDT per month, reduced duration (≤1 min) of teeth cleaning, teeth cleaning before breakfast, spoon feeding in early life, long-term (≥25 months) breastfeeding, and parental caries.

## 4. Discussion

This cross-sectional study describes the prevalence and risk factors of dental caries in children’s oral health. The overall prevalence of dental caries was 82.7% of pediatric patients in Mymensingh of Bangladesh. The prevalence varied in urban, suburban, and rural areas, i.e., 68.9%, 83.9%, and 96.3%, respectively. A similar overall prevalence of dental caries was reported in China 85% and Saudi Arabia 89% [28,55], and a comparatively lower prevalence was reported in Palestine (76%), Israel (64.7%), and India (51.9%) [24,25,56]. However, in high-income countries, the prevalence of dental caries is much lower and varies between 30% and 50% [57,58,59]. This difference is probably due to the higher level of education, the increased level of awareness, and improved accessibility to the dentist in developed country. Socioeconomic factors such as low family income can significantly increase the threat of dental caries development, which is supported by various previous studies. With the increase in family income, there is a decrease in the occurrence of dental caries [60]. A poor family financial status is related to limited access to oral-health services among these children [61,62,63]. The analysis also showed that children in a family whose parents earn ≤BDT 20,000/month were 4.75 times more vulnerable to dental caries. It is very likely that these parents cannot afford a dentist for their children due to the costs incurred for the dental materials, the dentist, and the medicine. The difference in the prevalence of dental caries in different residential areas may be because of a lack of parental oral-health awareness, unhealthy eating habits and oral hygiene practices, the high cost of dental treatment, and the limited accessibility and availability of dental services in rural areas. Parents living in urban and suburban areas are relatively more educated and economically solvent than those in rural areas of Bangladesh (personal observation). Educated people are generally more aware about oral hygiene and are more economically capable. Mothers who are educated to a higher level remain closer to their children and take oral hygiene more seriously [64,65,66]. In Bangladesh, dentist chambers are common in urban areas. Hence, dentists in urban areas are more accessible to parents. The risk factors analysis also revealed that children in rural areas are 7.3 times more vulnerable to dental caries than those in urban areas, which is supported by various previous studies [67,68,69,70,71]. Furthermore, the observed difference in dental caries may result from differences in socioeconomic factors, intake behaviors, and lifestyle conditions between rural and urban communities.

In the present study, there was no difference in the prevalence of dental caries between male and female (*p* > 0.05) children. These data are consistent with the results of Bullappa et al., 2017 [72]. However, the results are not in agreement with various other studies. In certain studies, there was a predominance of males over females [73,74,75], and in other studies, females over the males [46,76]. Thus, it is interfered that the difference in sex is not an important matter for dental caries. This may be because, at an early age, dietary and oral hygiene practices related to dental caries are mostly controlled by parents.

We observed a strong association with dental caries and the frequency and duration of teeth cleaning and teeth cleaning materials (*p* < 0.001). The American Dental Association (ADA) recommends that individuals brush twice per day and use floss or use other interdental cleaners once per day to effectively remove microbial plaques, which decrease caries development [77]. Similar findings were also reported in various other studies [33,46,78,79,80,81]. In Bangladesh, rural children are very reluctant to clean their teeth, and even if they do, they often do not use dental materials. This is because their parents are usually not educated in oral hygiene and are less capable of providing the dental materials.

This study revealed that there is a significant difference between the different feeding patterns in the early life of children, e.g., children who breastfed for <24 months were more prone to dental caries (*p* < 0.001). A similar conclusion was reported in various other studies [82,83,84,85,86,87,88,89]. Generally, children breastfeed before they sleep, and even sometimes while sleeping. During sleeping mode, the child’s mouth is not cleaned with water nor does it swallow milk properly. This residual milk in the mouth cavity may provide extra nutrients for the growth of micro-organisms, which, in turn, may influence the risk of dental caries.

There may be a genetic influence on the occurrence of dental caries. Our study revealed that children who have a familial history of dental caries are more at risk (OR 8.2) of dental caries. A similar association was reported by various other authors [90,91,92]. A correlation of dental decay in siblings was observed by Klein and Palmer (1938) [93]. Our study also revealed that dental caries prevalence is significantly lower in children who clean their teeth two times a day, e.g., after breakfast and before sleep at night. Brushing and cleaning of the mouth after eating removes residual food particles from the oral cavity and interdental spaces, which, in turn, reduces food fermentation and microbial growth. The present study was a hospital-based study, the findings of which may not reflect the population as a whole. Genetic factors, exposure to fluoride, oral biofilms, and water quality were not investigated in this study, all of which might have an influence on the occurrence of dental caries. Moreover, as it was a cross-sectional study, it was not possible to assess the temporality of exposure and outcome. Therefore, the findings from the present study should be used with caution. However, in this study, respondents were selected through randomization to minimize any selection bias. It would be worthwhile to carry out a population-based case-control study considering rural and urban settings. Moreover, awareness programs for parents, primary school teachers, and students concerning the risk of dental caries and good practices of oral hygiene should be introduced.

## 5. Conclusions

It was found that dental caries was the most common oral and periodontal disease in children in Mymensingh. Duration of breastfeeding (>24 months), time (only before breakfast in the morning) and duration of teeth cleaning (cleaning <1 min), spoon and bottle feeding in early life, history of family oral problems, parental residence in a rural area, and income of BDT ≤20,000 were identified as significant risk factors. Appropriate strategies based on the identified risk factors and dissemination of information through different public and private print and electronic media could play a role in reducing dental caries in children.

## Figures and Tables

**Table 1:** Prevalence of dental caries in relation to sociodemographic characteristics.

Variables		No. of Children Examined (*n* = 362)	No. of Cases (%)	95% CI
Age (Years)	4–7	266	218 (82.0 ^a^)	77.4–86.6
	8–10	96	82 (85.4 ^a^)	78.3–92.5
Sex	Male	197	165 (83.8 ^a^)	78.7–88.9
	Female	165	135 (81.8 ^a^)	75.9–87.7
Residence	Urban	135	93 (68.9 ^a^)	63.3–74.5
	Rural	134	129 (96.3 ^b^)	93.1–99.5
	Suburban	93	78 (83.9 ^c^)	76.4–91.4
Religion	Islam	315	266 (84.4 ^a^)	80.4–88.4
	Hindu	40	30 (75.0 ^a,b^)	61.6–88.4
	Christian	1	1 (100 ^a^)	-
	Buddhist	6	3 (50.0 ^b^)	9.9–90.0
Parental occupation	Daily labor	75	70 (93.3 ^a^)	90.3–96.3
	Cultivation	41	39 (95.1 ^a^)	92.5–97.7
	Livestock farming	16	12 (75.0 ^b^)	69.8–80.2
	Civil servant	56	41 (73.2 ^b^)	67.9–78.5
	Private organization	92	74 (80.4 ^b^)	75.6–85.2
	Own business	82	64 (78.0 ^b^)	73.0–83.0
Parental income (taka/month)	≤20,000	217	205 (94.5 ^a^)	91.5–97.5
	>20,000	145	95 (65.5 ^b^)	57.8–73.2
Maternal education level	Illiterate	77	77 (100 ^a^)	-
	Primary	131	122 (93.1 ^b^)	88.7–97.4
	SSC	73	69 (94.5 ^b^)	89.2–99.7
	HSC	28	11 (39.3 ^c,d^)	21.2–57.4
	Graduate	35	18 (51.4 ^d^)	34.8–67.9
	Post-graduate	18	3 (16.7 ^c^)	0.5–33.9
**Total**		**362**	**300 (82.7)**	

Values with different superscripts differ significantly (*p* < 0.05) within the variable under assessment. SSC = secondary school certificate; HSC = higher secondary certificate.

**Table 2:** Prevalence of dental caries in relation to dental practices.

Variables		No. of Children Examined (*n* = 362)	No. of Cases (%)	95% CI
Habit of teeth cleaning	Yes	359	297 (82.7 ^a^)	78.8–86.6
	No	3	3 (100 ^a^)	-
Teeth cleaning instrument	Tooth brush	255	200 (78.4 ^a^)	73.5–83.4
	* Miswak	4	4 (100 ^a^,^b^)	-
	Finger	42	42 (100 ^b^)	-
	Tooth brush + miswak + finger	58	51 (87.9 ^a^)	83.9–91.8
Teeth cleaning material	Tooth paste	148	103 (69.6 ^a^)	62.5–76.5
	Tooth powder	157	146 (93.0 ^b^)	89.1–96.9
	Tooth powder + charcoal	35	34 (97.1 ^b^)	94.5–99.7
	Toothpaste + toothpowder + charcoal	19	14 (73.7 ^a^)	66.9–80.4
Frequency of teeth cleaning (times per day)	Once	270	237 (87.8 ^a^)	83.9–91.4
	≥Twice	89	60 (67.4 ^b^)	61.8–73.0
Teeth cleaning duration	<1 min	50	48 (96.0 ^a^)	90.6–101.4
	1 min	62	57 (91.9 ^a^)	85.1–98.7
	2 min	69	44 (63.8 ^b^)	52.5–75.1
	Not definite time	135	118 (87.4 ^a^)	81.8–92.9
	>2 min	43	30 (69.8 ^b^)	56.1–83.5
Habit of tongue cleaning	Yes	31	18 (58.1 ^b^)	40.7–75.5
	No	331	282 (85.2 ^a^)	81.4–89.0
Habit of teeth flossing	Yes	36	27 (73.0 ^a^)	67.7–78.3
	No	326	273 (84.0 ^a^)	79.6–88.4
Flossing tool	Traditional tool	31	27 (87.1 ^a^)	75.3–98.9
	Professional floss	5	0 (0.0 ^b^)	-
Frequency of teeth flossing	Once	4	3 (75.0 ^a^)	32.5–117.4
	Twice	1	1 (100 ^a^)	-
	Occasionally	31	23 (74.2 ^a^)	58.8–89.6
Teeth cleaning period	Before breakfast	248	232 (93.5 ^a^)	90.5–96.5
	Whenever remember	54	38 (70.4 ^b^)	64.9–75.9
	Before breakfast + Before sleep at night	30	24 (80.0 ^a,b^)	75.2–84.8
	After breakfast + Before sleep at night	27	3 (11.1 ^c^)	7.3–14.9
Parental oral hygiene knowledge	Yes	98	62 (63.3 ^b^)	53.8–72.8
	No	264	238 (90.2 ^a^)	86.6–93.8
Parental supervision in teeth cleaning	Yes	149	113 (75.8 ^b^)	68.9–82.7
	No	213	187 (87.8 ^a^)	83.4–92.2
Frequency of parental supervision	Everyday	36	22 (61.1 ^a^)	45.2–77.0
	Weekly	12	8 (66.7 ^a,b^)	40.0–93.4
	Sometimes	101	83 (82.2 ^b^)	74.7–89.7
Family oral problem	Yes	190	177 (93.2 ^b^)	89.6–96.7
	No	172	123 (71.5 ^a^)	64.7–78.2

Values with different superscripts differ significantly (*p* < 0.05) within the variable under assessment. * Miswak = tooth-cleaning stick made from the roots, twigs, and stem of Salvadorapersica, Azadirachtaindica, and so on [54].

**Table 3:** Prevalence of dental caries in relation to eating and dietary habits.

Variables		No. of Children Examined (*n* = 362)	No. of Cases (%)	95% CI
Habit of eating chocolate	Yes	341	281 (82.4 ^a^)	78.4–86.4
	No	21	19 (90.5 ^a^)	77.9–103.0
Frequency of eating chocolate	Everyday	231	193 (83.5 ^a^)	78.7–88.3
	Weekly	18	15 (83.3 ^a^)	66.1–100.5
	Occasionally	92	73 (79.3 ^a^)	71.0–87.6
Habit of eating chips	Yes	350	288 (82.3 ^a^)	78.3–86.3
	No	12	12 (100 ^a^)	-
Frequency of eating chips	Everyday	227	188 (82.8 ^a^)	77.8–87.7
	Weekly	31	29 (93.5 ^a^)	84.8–102.2
	Occasionally	92	71 (77.2 ^a^)	68.6–85.8
Habit of drinking soft drinks	Yes	330	269 (81.5 ^b^)	77.3–85.7
	No	32	31 (96.9 ^a^)	90.8–102.9
Frequency of drinking soft drinks	Everyday	132	92 (69.7 ^a^)	61.8–77.5
	Weekly	44	40 (90.9 ^b^)	82.4–99.3
	Occasionally	154	137 (89.0 ^b^)	84.1–93.9
Habit of chewing gum	Yes	266	233 (87.6 ^b^)	83.6–91.6
	No	96	67 (69.8 ^a^)	60.6–78.9
Frequency of chewing gum	Everyday	79	69 (87.3 ^a^)	79.9–94.6
	Weekly	42	39 (92.9 ^a^)	85.1–100.6
	Occasionally	145	125 (86.2 ^a^)	82.1–90.3
Habit of eating biscuits	Yes	352	293 (83.2 ^a^)	79.3–87.1
	No	10	7 (70.0 ^a^)	41.6–98.4
Frequency of eating biscuits	Everyday	326	273 (83.7 ^a^)	79.7–87.7
	Weekly	5	4 (80.0 ^a^)	44.9–115.1
	Occasionally	21	16 (76.2 ^a^)	57.9–94.4
Source of chocolate, chips, soft drinks, chewing gum, biscuits	Family shop	66	63 (95.5 ^b^)	90.5–100.5
	Others	296	237 (80.1 ^b^)	75.6–84.6
Habit of drinking milk	Yes	323	273 (84.5 ^b^)	75.7–84.5
	No	39	27 (69.2 ^a^)	54.7–83.7
Properties of drinking milk	With sugar	250	227 (90.8 ^a^)	87.2–94.4
	Without sugar	73	46 (63.0 ^b^)	51.9–74.1
Frequency of drinking milk	Everyday	236	208 (88.1 ^a^)	83.9–92.2
	Weekly	11	8 (72.7 ^a,b^)	46.4–99.0
	Occasionally	76	57 (75.0 ^b^)	65.3–84.7
Feeding methods during early life	Bottle feeding	196	161 (82.1 ^a^)	76.7–87.5
	Spoon feeding	136	119 (87.5 ^a^)	83.5–91.5
	None	30	20 (66.7 ^b^)	61.0–72.4
Properties of food stuff during early life	Milk without sugar	44	9 (21.4 ^a^)	9.3–33.5
	Milk with sugar	98	91 (92.9 ^b^)	87.8–97.9
	Blended food (rice, meat, fish, vegetable, suji)	174	164 (94.3 ^b^)	91.5–97.1
	Packaged food (dry foods, oats, fruits juice)	16	16 (100 ^b^)	-
Habit of breastfeeding in early life	Yes	345	293 (84.9 ^b^)	81.1–88.7
	No	17	7 (41.2 ^a^)	17.8–64.6
Duration of breastfeeding (months)	0–24	110	69 (62.2 ^a^)	56.4–68.0
	25–36	115	106 (92.2 ^b^)	87.3–97.1
	37–48	87	80 (98.9 ^c^)	96.7–101.1
	≥49	33	32 (97.1 ^b,c^)	95.1–99.1

Values with different superscripts differ significantly (*p* < 0.05) within the variable under assessment.

**Table 4:** Sociodemographic factors * associated with the occurrence of dental caries in pediatric patients.

Variables	Categories	Odds Ratio (OR)	95% CI	*p*-Value
Age (Years)	4–7	Reference		
	8–10	1.29	0.675–2.464	0.441
Sex	Male	1.15	0.663–1.981	0.626
	Female	Reference		
Residence	Urban	Reference		
	Rural	11.65	4.44–30.58	<0.001
	Suburban	2.35	1.21–4.56	0.01
Religion	Islam	5.43	1.07–27.68	0.04
	Hindu	3.00	0.52–17.32	0.22
	Christian	Undetermined	-	-
	Buddhist	Reference		
Parental occupation	Daily labor	3.91	1.38–11.22	0.01
	Cultivation	5.48	1.21–24.93	0.03
	Livestock farming	0.84	0.24–2.94	0.79
	Civil servant	0.79	0.35–1.69	0.51
	Private organization	1.16	0.56–2.41	0.70
	Own business	Reference		
Parental income (per month)	≤20,000	8.99	4.58–17.67	<0.001
	>20,000	Reference		
Maternal education level	Illiterate	Undetermined	-	-
	Primary	67.78	16.51–278.03	<0.001
	SSC	86.25	17.45–426.21	<0.001
	HSC	3.24	0.76–13.84	0.11
	Graduate	5.29	1.29–21.59	0.02
	Post-Graduate	Reference		

* Factors were identified by bivariable logistic regression.

**Table 5:** Dietary and oral hygiene practices * associated with the occurrence of dental caries in pediatric patients.

Variables	Categories	Odds Ratio (OR)	95% CI	*p*-Value
Habit of teeth cleaning	Yes	Reference		
	No	Undetermined	-	-
Teeth cleaning instrument	Tooth brush	Reference		
	Miswak	Undetermined	-	-
	Finger	Undetermined	-	-
	Toothbrush + miswak + finger	2.00	0.32–3.69	0.11
Teeth cleaning material	Tooth paste	Reference		
	Tooth powder	5.28	2.60–10.72	<0.001
	Tooth powder + charcoal	14.85	1.97–111.89	0.01
	Tooth paste + toothpowder + charcoal	1.22	0.42–3.60	0.71
Teeth cleaning frequency	Once	3.47	1.96–6.16	<0.001
	≥Twice	Reference		
Teeth cleaning duration	<1 min	10.4	2.19–49.34	0.003
	1 min	4.9	1.61–15.17	0.01
	2 min	0.76	0.34–1.72	0.52
	Not definite time	3.00	1.32–6.87	0.01
	>2 min	Reference		
Habit of tongue cleaning	Yes	Reference		
	No	4.16	1.92–9.02	<0.001
Habit of teeth flossing	Yes	Reference		
	No	1.72	0.76–3.86	0.19
Flossing tool	Traditional	Undetermined	-	-
	Professional	Reference		
Flossing frequency	Once	Reference		
	Twice	Undetermined	-	-
	Occasionally	0.96	−0.21–2.13	0.97
Teeth cleaning period	Before breakfast	116	31.53–426.82	<0.001
	Whenever remember	19	5.00–72.19	<0.001
	Before breakfast + Before sleep at night	32	7.16–142.98	<0.001
	After breakfast + Before sleep at night	Reference		
Parental oral hygiene knowledge	Yes	Reference		
	No	5.32	2.99–9.46	<0.001
Parental supervision in teeth cleaning	Yes	Reference		
	No	2.29	1.31–3.99	0.003
Frequency of parental supervision	Everyday	Reference		
	Weekly	1.27	0.32–5.03	0.73
	Sometimes	2.93	1.26–6.81	0.01
Habit of eating chocolate	Yes	0.49	0.11–2.17	0.35
	No	Reference		
Frequency of eating chocolate	Everyday	1.32	0.72–2.44	0.37
	Weekly	1.30	0.34–4.96	0.70
	Occasionally	Reference		
Habit of eating chips	Yes	Undetermined	-	-
	No	Reference		
Frequency of eating chips	Everyday	1.43	0.79–2.59	0.24
	Weekly	4.29	0.94–19.48	0.06
	Occasionally	Reference		
Habit of drinking soft drinks	Yes	0.14	0.02–1.06	0.06
	No	Reference		
Frequency of drinking soft drinks	Everyday	0.29	0.15–0.53	<0.001
	Weekly	1.24	0.39–3.89	0.71
	Occasionally	Reference		
Habit of chewing gum	Yes	3.06	1.73–5.39	<0.001
	No	Reference		
Frequency of chewing gum	Everyday	1.10	0.49–2.49	0.81
	Weekly	2.08	0.59–7.37	0.26
	Occasionally	Reference		
Habit of eating biscuits	Yes	2.13	0.54–8.47	0.28
	No	Reference		
Frequency of eating biscuits	Everyday	1.61	0.57–4.58	0.37
	Weekly	1.25	0.11–13.92	0.86
	Occasionally	Reference		
Source of chocolate, chips, soft drinks, chewing gum, biscuits	Family shop	5.23	1.59–17.23	0.01
	Others	Reference		
Habit of drinking milk	Yes	Reference		
	No	0.41	0.196–0.867	0.02
Properties of drinking milk	With sugar	5.79	3.05–10.98	<0.001
	Without sugar	Reference		
Frequency of drinking milk	Everyday	Reference		
	Weekly	0.36	0.09–1.43	0.15
	Occasionally	0.40	0.21–0.76	0.01
Feeding methods during early life	Bottle	2.30	0.99–5.34	0.05
	Spoon	3.50	1.40–8.73	0.007
	None	Reference		
Properties of food stuff during early life	Without sugar	Reference		
	With sugar	50.55	17.48–146.19	<0.001
	Blender food	63.77	24.14–168.52	<0.001
	Packaged food	Undetermined	–	–
Habit of breastfeeding in early life	Yes	Reference		
	No	0.124	0.05–0.34	<0.001
Duration of breastfeeding (months)	0–24	Ref		
	25–36	6.99	3.2–15.31	<0.001
	37–48	51.1	6.86–380.95	<0.001
	≥49	19.01	2.5–144.42	0.004
Family oral problem	Yes	5.42	2.82–10.42	<0.001
	No	Reference		

* Factors were identified by bivariable logistic regression.

**Table 6:** Potential risk factors of dental caries in multiple logistic regression.

Variables	Categories	Odds Ratio (OR)	95% CI	*p*-Value
Residence	Urban	Reference		
	Rural	7.31	1.73–30.83	0.01
	Suburban	0.86	0.25–2.95	0.81
Parental income	≤20,000	4.75	1.49–15.05	0.01
	>20,000	Reference		
Teeth cleaning duration	<1 min	10.08	1.02–99.88	0.05
	1 min	18.54	2.05–168.17	0.01
	2 min	1.43	0.29–7.08	0.66
	Not definite time	3.08	0.67–14.22	0.15
	>2 min	Reference		
Feeding method in early life	Bottle	10.81	1.71–68.30	0.01
	Spoon	12.57	2.09–75.61	0.01
	None	Reference		
Duration of breastfeeding (months)	0–24	Reference		
	25–36	14.19	3.88–51.90	<0.001
	37–48	212.53	8.69–5195.25	0.001
	≥49	28.09	1.63–485.75	0.02
Family oral problem	Yes	8.20	2.57–26.16	<0.001
	No	Reference		
Teeth cleaning period	Before breakfast	93.3	10.95–795.32	<0.001
	Whenever remember	17.79	1.99–158.72	0.01
	Before breakfast + before sleep at night	75.46	5.98–952.05	0.001
	After breakfast + before sleep at night	Reference		

## Data Availability

All the data are made available here in the manuscript.
